# Elevated Levels of Organochlorine Pesticides in South Asian Immigrants Are Associated With an Increased Risk of Diabetes

**DOI:** 10.1210/js.2017-00480

**Published:** 2018-05-22

**Authors:** Sarah I Daniels, John C Chambers, Sylvia S Sanchez, Michele A La Merrill, Alan E Hubbard, Anthony Macherone, Matthew McMullin, Luoping Zhang, Paul Elliott, Martyn T Smith, Jaspal Kooner

**Affiliations:** 1 Superfund Research Center, School of Public Health, University of California, Berkeley, California; 2 Lee Kong Chian School of Medicine, Nanyang Technological University, Singapore, Singapore; 3 Department of Epidemiology and Biostatistics, Imperial College, London, United Kingdom; 4 Department of Cardiology, Ealing Hospital, Middlesex, United Kingdom; 5 Imperial College Healthcare NHS Trust, London, United Kingdom; 6 MRC-PHE Centre for Environment and Health, Imperial College, London, United Kingdom; 7 Department of Environmental Toxicology, University of California, Davis, California; 8 Agilent Technologies, Inc., Santa Clara, California; 9 The Johns Hopkins University School of Medicine, Baltimore, Maryland; 10 NMS Laboratories, Willow Grove, Pennsylvania; 11 National Heart and Lung Institute, Faculty of Medicine, Imperial College, London, United Kingdom

**Keywords:** dichlorodiphenyldichloroethylene (DDE), dichlorodiphenyltrichloroethane (DDT), hexachlorohexane, India, persistent organic pollutants, Sri Lanka

## Abstract

**Objective:**

Rates of diabetes mellitus are higher in South Asians than in other populations and persist after migration. One unexplored cause may be higher exposure to persistent organic pollutants associated with diabetes in other populations. We compared organochlorine (OC) pesticide concentrations in South Asian immigrants and European whites to determine whether the disease was positively associated with OC pesticides in South Asians.

**Research Design and Methods:**

South Asians of Tamil or Telugu descent (n = 120) and European whites (n = 72) were recruited into the London Life Sciences Population Study cohort. Blood samples as well as biometric, clinical, and survey data were collected. Plasma levels of *p,p′*-dichlorodiphenyldichloroethylene (DDE), *p,p′*- dichlorodiphenyltrichloroethane, *β*-hexachlorohexane (HCH), and polychlorinated biphenyl-118 were analyzed by gas chromatography-mass spectrometry. South Asian cases and controls were categorized by binary exposure (above vs below the 50th percentile) to perform logistic regression.

**Results:**

Tamils had approximately threefold to ninefold higher levels of OC pesticides, and Telugus had ninefold to 30-fold higher levels compared with European whites. The odds of exposure to *p,p′*-DDE above the 50th percentile was significantly greater in South Asian diabetes cases than in controls (OR: 7.00; 95% CI: 2.22, 22.06). The odds of exposure to *β*-HCH above the 50th percentile was significantly greater in the Tamil cases than in controls (OR: 9.35; 95% CI: 2.43, 35.97).

**Conclusions:**

South Asian immigrants have a higher body burden of OC pesticides than European whites. Diabetes mellitus is associated with higher *p,p′*-DDE and *β*-HCH concentrations in this population. Additional longitudinal studies of South Asian populations should be performed.

## Teaser

We found blood levels of OC pesticides in South Asian immigrants to the United Kingdom were much higher than in European whites and were associated with a sevenfold increased rick of diabetes.

Over 80 million adults are living with diabetes mellitus in India (9% to 10% prevalence), and approximately 90% of these adults have type 2 diabetes [1]. Rates of type 2 diabetes are also high in South Asian diaspora populations, including those in the United Kingdom [2–4]. South Asians living in the United Kingdom have a twofold to threefold higher rate of type 2 diabetes than European whites [2–4]. Diabetes mellitus develops in South Asian Indians at a lower body weight, blood lipid level, and age than in other ethnic groups, yet known risk factors, including genetics, do not explain this increased vulnerability [5, 6]. One possibility is that South Asians have a higher exposure to organochlorine (OC) pesticides, which have been associated with diabetes mellitus in European, American, and Korean populations [7].

South Asians have been exposed to OC pesticides for longer periods and at higher concentrations than populations in Western Europe, where these legacy compounds were largely phased out in the 1970s and 1980s. For example, unregulated spraying of *p,p′*- dichlorodiphenyltrichloroethane (DDT) and lindane [*γ*-hexachlorohexane (*γ*-HCH)] for control of mosquito-borne diseases and agricultural purposes continued in India until after ratification of the Stockholm Convention in 2006 [8, 9]. Today, India is still the top producer and consumer of OC pesticides [10, 11] and has some of the world’s highest-recorded breast milk concentrations of these pesticides, including DDT and HCH [12, 13]. Unlike in other Asian nations such as China, environmental levels of DDT and HCHs in India have not appeared to decrease since the implementation of tighter regulations [8].

DDT and dichlorodiphenyldichloroethylene (DDE) are stored for long periods in body fat and are resistant to metabolism, with plasma half-lives of 2 and 6 to 7 years, respectively, in humans [14]. Lindane is fairly short-lived in the environment and humans [9], but the *β*-isomer (*β*-HCH), an impurity formed during production of lindane, has a half-life of 7 years in humans [15]. Hence, these OC pesticides should persist in the bodies of South Asian immigrants many years after they migrate to the West. We therefore measured blood levels of two subclasses of persistent organic pollutants (POPs), OC pesticide derivatives and polychlorinated biphenyls (PCBs), in subjects recruited for The London Life Sciences Prospective Population Study (LOLIPOP), a cohort that includes South Asian immigrants and European whites residing in the London area. Several genetic and epigenetic studies of diabetes susceptibility have been performed previously on the LOLIPOP cohort [5, 16, 17], but environmental exposures associated with diabetes mellitus have not yet been assessed in this population. PCBs were considered a negative control in this study, as this chemical class of analytes was not predicted to have differing levels within the sample population. We hypothesized that (1) baseline levels of OC pesticides are higher in South Asian immigrants than in European whites in the London area and (2) diabetes mellitus is associated with OC pesticide exposure in South Asians.

## 1. Subjects and Methods

### A. Study Population and Design

The LOLIPOP cohort comprised >30,000 South Asians and European whites living in West London [18]. The subjects in the current study were adult volunteers (>21 years of age) of mostly Telugu or Sri Lankan Tamil descent who were newly recruited into the LOLIPOP cohort in 2012. The study was approved by the UK National Research Ethics Service (07/H0712/150) and by the Berkeley Committee for Protection of Human Subjects. During their enrollment, the subjects completed a questionnaire in which they were asked if they had a medical history of diabetes and when it was diagnosed. An additional six new diabetic cases were identified by fasting plasma glucose (FPG) level ≥7 mmol/L at the time of blood collection. These six case diagnoses were based on a single blood collection. Nondiabetic controls were defined as having no history of diabetes and an FPG level <5.6 mmol/L. None of the subjects with a prior diagnosis of diabetes had an FPG level <5.6 mmol/L. FPG was chosen instead of hemoglobin A1c primarily to classify diabetes cases because the latter has had low sensitivity in Asians [19].

We examined a total of 192 individuals: 120 South Asians of Indian Telugu or Sri Lankan Tamil descent and 72 European whites (Table 1). The Tamils migrated to the United Kingdom before the Telugus did (an average of 20 years vs 12 years before recruitment for Tamils and Telugus, respectively). The European whites were born in the United Kingdom. Among the 120 recruited South Asians, there were 24 cases of diabetes mellitus (four Telugus, 20 Tamils) and 96 nondiabetic controls (43 Telugus, 53 Tamils). We assumed that the 24 diabetes mellitus cases were almost all patients with type 2 diabetes, but it is possible some patients had type 1, although the prevalence of the latter in LOLIPOP is very low. The South Asian controls were frequency matched to the diabetes cases on age, sex, proportion of Telugus, smoking status, and waist-hip ratio (Table 1). The South Asian control group was also frequency matched to a European white comparison group (n = 72 controls) on similar characteristics.

**Table 1.** Characteristics of the Study Subjects

	Whites	South Asians		
	Controls	Controls	Cases	Cases vs Controls
	n = 72	n = 96	n = 24	*P* Value
Males, %	36 (50)	53 (55.2)	17 (70.8)	0.25
Smoke, %	12 (16.7)	4 (4.17)	3 (12.5)	0.14
Drink, %	43 (59.7)	25 (26.0)	5 (20.8)	0.79

	Mean	SD	Mean	SD	Mean	SD	*P* Value
Age, y	48.49	6.65	48.32	8.38	56.10	9.57	<0.01
SBP, mm Hg	122.00	15.1	123.90	13.50	131.75	15.15	0.02
DBP, mm Hg	76.70	10.6	78.56	9.13	81.17	7.79	0.15
BMI, kg/m^2^	26.43	4.55	26.52	3.37	26.14	3.53	0.63
WHR	0.89	0.08	0.93	0.07	0.97	0.07	0.01
HDL, mmol/L	1.54	0.46	1.32	0.32	1.23	0.36	0.28
LDL, mmol/L	3.02	0.84	3.36	0.84	2.16	1.08	<0.01
Glucose, mmol/L	4.97	0.34	4.83	0.33	9.02	1.63	<0.01
% HbA1c	5.41	0.30	5.62	0.40	8.20	1.29	<0.01
Chol, mg/dL	195.30	34.2	204.22	35.35	165.82	50.94	<0.01
Trig, mg/dL	99.40	59.9	120.31	56.42	167.78	83.12	0.01
Years in the United Kingdom*^a^*			16.33	11.28	23.96	12.06	0.01
Body fat, %	30.88	8.70	32.34	7.75	29.61	7.89	0.14

Abbreviations: BMI, body mass index; Chol, cholesterol; DBP, diastolic blood pressure; HbA1c, hemoglobin A1c; HDL, high-density lipoprotein; LDL, low-density lipoprotein; SBP, systolic blood pressure; Trig, triglycerides; WHR, waist/hip ratio.

^a^ All white control subjects were born in the United Kingdom.

### B. Chemical Analysis

Plasma was extracted in four batches using chemical denaturation, liquid-liquid extraction, solid-phase cleanup, and reconstitution with hexanes. An Agilent 7890B gas chromatograph coupled to an Agilent 7000C GC Triple Quadrupole mass spectrometer was operated in electron ionization, multiple-reaction monitoring mode. System performance was monitored with calibrators at seven concentration levels for each analyte. Variability was measured using pooled reference samples at several intervals within each batch run.

We report measurements of the POPs *p,p′*-DDE, *p,p′-*DDT, *β*-HCH, and PCB-118 in the plasma of 192 participants. Several other PCBs were also measured, including PCB-123, PCB-114, PCB-105, PCB-167, PCB-156, PCB-157, and PCB-189; however, these PCBs were detected in only some of the samples. Among the measured PCBs, only PCB-118 was above the limit of detection and limit of quantitation for a majority of the samples and hence was used as the PCB analyte of choice. Concentrations (ng/mL) were converted to lipid-adjusted values by using the available clinical lipid profile measurements and the following formula: Total lipids = (2.27 × total cholesterol) + triglyceride + 0.623 [20].

### C. Statistical Analysis

The Wilcoxon rank sum test was used to examine baseline differences in chemical concentrations within and between South Asian controls (n = 96) and European whites (n = 72). To assess the association between individual chemicals and diabetes mellitus within the South Asian sample population, we created two groups according to whether each chemical concentration was above or below the median concentration. Logistic regression was used to obtain unadjusted ORs for concentrations above vs below the median value. Small sample size adjustments for ORs were calculated instead of logistic regression where appropriate. *P* values for the 2 × 2 contingency tables of case/control and above/below median POP concentrations were obtained using the Fisher’s exact *t* test. Multivariable models included adjustments for age, sex, waist/hip ratio, systolic blood pressure, smoking status (yes/no), and alcohol use (yes/no).

## 2. Results

Significantly higher concentrations of organochloring pesticides were observed in South Asian immigrants than in European whites living in West London. Although some difference was expected, the median concentrations of *p,p′*-DDE and *p,p′*-DDT among nondiabetic control participants were more than eightfold higher (*P* < 0.001) in South Asians [median: 535.87 ng/g-lipid (range: 26.82 to 25,143.8 ng/g-lipid) and median: 17.65 ng/g-lipid (range: 3.91 to 316.45 ng/g-lipid) for *p,p′*-DDE and *p,p′*-DDT, respectively] than in whites [median: 61.26 ng/g-lipid (range: 17.65 to 353.3 ng/g-lipid) and median: 2.08 ng/g-lipid (range: 0.64 to 70.97 ng/g-lipid) for *p,p′*-DDE and *p,p′*-DDT, respectively]. This eightfold difference is large considering that most of the South Asian immigrants had been living in London for many years. As expected, PCB-118 concentrations were similar (*P* = 0.51) in the two groups [median: 4.51 ng/g-lipid (range: 0.81 to 34.21 ng/g-lipid) and median: 3.94 ng/g-lipid (range: 0.89 to 13.38 ng/g-lipid) for South Asians and whites, respectively].

Unlike with *p,p′*-DDE, *p,p′*-DDT, and PCB-118, the baseline distribution of *β*-HCH concentrations differed widely by South Asian ethnic group. Median *β*-HCH levels were threefold higher (*P* < 0.001) in Tamil control individuals [median: 36.73 ng/g-lipid (range: 4.63 to 541.67ng/g-lipid)] and 30-fold higher (*P* < 0.001) in Telugus [median: 365.32 ng/g-lipid (range: 96.86 to 714.45 ng/g-lipid)] than in whites [median: 12.86 ng/g-lipid (range: 3.18 to 36.44 ng/g-lipid)]. Similar fold-change differences were found when levels were expressed in ng/mL units.

Significant associations were found between OC pesticides and diabetes mellitus in South Asians. We observed sevenfold increased odds of *p,p′*-DDE plasma concentrations occurring above the median in diabetes mellitus cases compared with controls [OR: 7.00 (95% CI: 2.22, 22.06)] (Table 2). The OR for *p,p′*-DDT binary exposure was not significantly different between cases and controls. A significant association for PCB-118 was also found in ng/g-lipid units [OR: 2.99 (95% CI: 1.13, 7.88)] but could not be replicated using ng/mL units (Table 3). The Tamils and Telugus had widely differing exposure levels of *β*-HCH, and so inferences could not be based on binary exposure levels across the entire South Asian group. Thus, South Asians were stratified into Tamil and Telugu subgroups before associations between *β*-HCH levels and diabetes mellitus were assessed. Significantly increased odds [OR: 9.35 (95% CI: 2.43, 35.97)] of *β*-HCH concentrations above the median was observed in Tamils with diabetes mellitus compared with controls (Table 4). For the smaller Telugu population, the OR was elevated but not significant [OR: 4.38 (95% CI: 0.52, 203.36)].

**Table 2.** POP Concentrations and Numbers Above/Below Median for South Asian Diabetic Cases vs Controls (ng/g-lipid)

		Controls		Cases			
Compound	Groups Split at Median	n	Median (Range)	n	Median (Range)	Odds Ratio (95% CI)*^a^*	*P* Value
*p,p'*-DDE	<710.87	56	318.00 (26.82, 705.10)	4	208.34 (141.38, 552.80)	7.00 (2.22, 22.06)	<0.001
	≥710.87	40	1282.48 (736.62, 25143.80)	20	1698.55 (716.627, 6212.58)		
*p,p'*-DDT	<17.61	47	11.12 (3.91, 17.57)	13	10.03 (6.24, 16.05)	0.8 (0.33, 1.99)	0.82
	≥17.61	49	30.91 (17.65, 316.50)	11	28.91 (17.66, 194.90)		
PCB-118	<4.36	53	2.66 (0.81, 4.33)	7	2.76 (2.03, 4.22)	2.99 (1.13, 7.88)	0.04
	≥4.36	43	7.33 (4.40, 34.21)	17	6.32 (4.52, 27.34)		

^a^ Adjustment for age, waist/hip ratio, sex, smoking status, and alcohol use did not change the effect size or significance levels except for PCB-118 for South Asians [OR_adj_ = 2.56 (95% CI: 0.80, 8.16)].

**Table 3.** POPs Concentrations and Numbers Above/Below Median for South Asian Diabetic Cases vs Controls (ng/mL)

		Controls		Cases			
Compound (ng/mL)	Exposure Status	n	Median (Range)	n	Median (Range)	Odds Ratio (95% CI)	*P* Value
*p,p'*-DDE	<3.82	54	1.86 (0.16, 3.76)	6	2.49 (1.18, 3.79)	5.01 (1.40, 17.99)	0.01
	>3.82	42	7.54 (3.84, 145.85)	18	12.11 (3.92, 52.99)		
*p,p'*-DDT	<0.11	47	0.07 (0.02, 0.11)	13	0.06 (0.03, 0.10)	0.94 (0.29, 3.08)	0.82
	>0.11	49	0.19 (0.11, 2.17)	11	0.24 (0.11, 1.34)		
PCB-118	<0.03	49	0.02 (0.006, 0.02)	11	0.02 (0.01, 0.02)	1.60 (0.52, 4.97)	0.82
	>0.03	47	0.05 (0.03, 0.24)	13	0.05 (0.03, 0.12)		

**Table 4.** *β*-HCH Concentrations and Numbers Above/Below Median for Diabetic Cases vs Controls in Tamil and Telugu Populations (ng/g-lipid)

			Controls		Cases			
Compound (ng/g-lipid)	Population	Groups Split at Median	n	Median (Range)	n	Median (Range)	Odds Ratio (95% CI)	*P* Value
*β*-HCH	Tamil	<50.58	33	27.12 (4.63, 48.98)	3	49.30 (35.61, 49.89)	9.35*^a^* (2.43, 35.97)	<0.001
		≥50.58	20	84.61 (50.58, 541.70)	17	95.35 (52.03, 499.20)		
	Telugu	<369.30	23	272.81 (96.86, 365.42)	0	N/A	4.38 (0.52, 203.36)	0.11
		≥369.30	20	461.41 (369.34, 714.45)	4	535.66 (374.28, 627.60)		

Abbreviation: N/A, not available.

^a^ Further adjustment for age, waist/hip ratio, sex, smoking status, and alcohol use did not widely change the effect size nor significance levels except for Tamils [OR_adj_ = 7.01 (95% CI: 1.44, 34.0)].

## 3. Discussion

Our study compared blood plasma POP levels of South Asians with those of European whites residing in the same Western city. Blood levels of various OC pesticides were much higher in the South Asian immigrants than in the whites born in the United Kingdom, whereas levels of PCBs were not significantly different. The differences in OC pesticide levels between South Asians and whites were sustained for at least 10 to 20 years after the South Asians had migrated to the relatively low-exposure UK environment. The predicted blood half-life for *p,p′*-DDE and *p,p′*-DDT is 6 to 7 years and 2 years, respectively [14], yet they were still detected in almost all of the participant samples. Lindane (*γ*-HCH) is fairly short-lived [9], but the *β*-HCH isomer (an impurity formed during production of lindane) has a half-life of 7 years [15]. Lindane was detected in only 75% of our participant samples, and the signal was 32-fold lower than that of *β*-HCH in the analytical data. In addition, the *β*-HCH levels varied greatly depending on country of origin (*i.e.,* Sri Lanka vs India), indicative of the varied use of HCHs across the Indian subcontinent. Within South Asian ethnic groups, Telugus had twofold higher levels of *p,p′*-DDT and eightfold to 10-fold higher levels of *β*-HCH than Tamils. The higher concentration of *p,p′*-DDT in Telugus may be due to their more recent exposure, as this subgroup migrated to the United Kingdom 10 years after the Tamils (on average). The higher *β*-HCH levels in Telugus may be due to more prevalent use of lindane in India than in Sri Lanka [11]. Overall, these findings of high but varying levels of OC pesticides in different ethnic groups are most likely the result of high exposure in early life in India or Sri Lanka and perhaps ongoing exposure from Indian foods, such as ghee [21], and are not due to differences in clearance of the compounds.

Significant associations between diabetes mellitus and *p,p′*-DDE and *β*-HCH levels were also found in the South Asians, with greater overall effect sizes than those generally reported in other studies [7]. Significant associations were found with *p,p′*-DDE and *β*-HCH in both lipid-adjusted (ng/g-lipid) and unadjusted (ng/mL) units. The lack of a strong association of diabetes mellitus with PCBs in this population demonstrates the specificity of this finding to OC pesticides. Associations between type 2 diabetes and *p,p′*-DDE have been observed in cross-sectional studies in Americans [22–24], Native Americans [25], Koreans [26], and Slovakians [27], as well as in prospective studies in Great Lakes fish consumers [28] and Swedes [29]. There are no prior studies of this association in a UK population. A link between *β*-HCH and preexisting type 2 diabetes has also been reported in cross-sectional studies involving Americans [22, 23], Koreans [26], Slovakians [27], Saudi Arabians [30], and Norwegians [31]. Again, there are no prior studies of this association in a UK population. Taken together, our results suggest that the high levels of OC pesticides found in South Asian immigrants may help explain their greater susceptibility to diabetes mellitus.

Our finding has potentially important implications for public health because disproportionate exposure to diabetes-associated endocrine disrupting chemicals (EDCs) may be an underappreciated contributor to disparities in metabolic disease risk. Ruiz *et al.* [32] described how the burden of diabetes is not uniformly borne in American society, as the disease disproportionately affects certain populations, including African Americans, Latinos, and low-income individuals. Among these susceptible populations, numerous studies have reported significantly higher exposures to diabetogenic EDCs, including OC pesticides. The presence of high levels of OC pesticides in South Asian migrants to the United Kingdom makes them a similar “at risk” population. Further, it suggests that immigrants may be “silent carriers” of high exposure who themselves may not be aware of prior high-exposure experiences and who may be surrounded by public and medical health communities that are also unaware of their increased risk. A future goal should be to perform further studies of the association between diabetes risk and EDC exposure in subpopulations, including migrant communities, and to educate the medical community about early-life EDC exposure as a risk factor for diabetes mellitus.

Animal and tissue culture models support the association with OC pesticides and provide additional evidence of mechanisms for glucose dysregulation and reduced insulin sensitivity from OC pesticide exposure. Associations between DDT exposure and blood glucose levels were initially found in rats [33] and mice [34] >40 years ago. More recent studies in mice have shown acute exposure to DDE increases fasting blood glucose levels and body weight for 7 to 21 days after treatment [35]. Another study in female mice showed that perinatal DDT exposure reduced core body temperature, impaired cold tolerance, decreased energy expenditure, and produced a transient early-life increase in body fat in female offspring [36]. When challenged with a high-fat diet for 12 weeks in adulthood, female offspring perinatally exposed to DDT developed glucose intolerance, hyperinsulinemia, dyslipidemia, and altered bile acid metabolism. Perinatal DDT exposure combined with high-fat feeding in adulthood further impaired thermogenesis as evidenced by reductions in core temperature and in the expression of numerous RNAs that promote thermogenesis and substrate utilization in the brown adipose tissue of adult female mice [36]. Hence, perinatal DDT exposure in mice impairs thermogenesis and the metabolism of carbohydrates and lipids, which may increase susceptibility to metabolic syndrome in adult female offspring. Similar results have been reported in rats [37]. These studies suggest that *in utero* and early-life exposure to DDT or DDE in children may predispose adults to the harmful effects of a high-fat Western diet. This may help explain the high preponderance of diabetes mellitus in immigrants who have migrated to the West from locations with a high utilization of DDT, such as India and Mexico.

Studies in cell culture also support the association. *In vitro,* pancreatic *β*-cells chronically exposed to *p,p′-*DDT or *p,p′-*DDE decreased protein expression involved in the hyperglycemia stress response [38]. In addition, glucose dysregulation has been observed following acute treatment with lindane in animal and cell models, yet the opposite effects were seen *in vitro* vs *in vivo* [39]. Although individual OC pesticides have been shown to affect glucose metabolism in experimental models, the combined effect of mixtures on metabolic changes needs further elucidation.

This study has several limitations. First, the small number of cases did not allow for rigorous statistical analysis in the Tamil and Telugu subgroups. However, the observed trends suggest that there are higher levels of OC pesticides in diabetes mellitus cases than in controls. The small number of cases also made it difficult to examine dose-response relationships in this study. However, we conducted exploratory analyses and calculated the odds of diabetes within tertile levels of POP exposure (Supplemental Table 1). CIs were broad as expected, and the OR estimate for each tertile was within the CI of the adjacent tertile. We were reassured that a dose response appeared evident for DDE. Second, the OC pesticide exposure measurements in this study were highly correlated (Supplemental Fig. 1). Thus, OC pesticides may not contribute independently to diabetes risk and could act through similar mechanisms. Third, diabetes risk may be more dependent on the timing and dose of cumulative OC pesticide levels as opposed to current measurements of single analytes or chemical classes. In the future, cohort studies on banked blood samples from the LOLIPOP and other studies, such as the Mediators of Atherosclerosis in South Asians Living in America cohort of South Asian migrants to the United States, could be used to demonstrate exposure-disease temporality.

Despite these limitations, this study adds to the growing literature of positive epidemiological associations between OC pesticides and diabetes mellitus. There have been few biomonitoring studies of OC pesticides in South Asian migrants to date and, to our knowledge, no studies examining the relationship between their high rates of diabetes mellitus and OC pesticide exposure. Future prospective studies on OC pesticides in Indians should focus on native and migrant South Asians, who historically have had high exposure to multiple pesticides and have a disproportionately high risk of developing diabetes. South Asians comprise a substantial proportion of the world’s population; thus, confirmation of the associations we found here between OC pesticides and diabetes mellitus could have public health implications on a global scale.

## Supplementary Material

Supplemental Data

## Abbreviations:

DDE: dichlorodiphenyldichloroethylene
DDT: dichlorodiphenyltrichloroethane
EDC: endocrine disrupting chemical
FPG: fasting plasma glucose
HCH: hexachlorohexane
LOLIPOP: London Life Sciences Prospective Population Study
NHS: National Health Service
NIHR: National Institute for Health Research
OC: organochlorine
PCB: polychlorinated biphenyl
POP: persistent organic pollutant
