# Supplementary material for: Elevated Levels of Organochlorine Pesticides in South Asian Immigrants Are Associated With an Increased Risk of Diabetes
Source: J Endocr Soc. 2018 May 22;2(8):832–41. doi: 10.1210/js.2017-00480 (PMC6041775; doi:10.1210/js.2017-00480)
Supplement: Supplemental Data [file js.2017-00480.sd1.docx]

Supplementary Table 1: Odds Ratios for T2D given exposure in either Tertile 2 or Tertile 3 vs. lowest tertile (Tertile 1)

|  | **Unadjusted** | | | | **Adjusted*** | | | |
| --- | --- | --- | --- | --- | --- | --- | --- | --- |
|  | **Tertile 2** | | **Tertile 3** | | **Tertile 2** | | **Tertile 3** | |
| **Compound (ng/g-lipids)** | **OR** | **95% CI** | **OR** | **95% CI** | **OR** | **95% CI** | **OR** | **95% CI** |
| DDE | 1.6 | (0.41, 6.15) | 5.6** | (1.67, 18.78) | 0.97 | (0.22, 4.21) | 3.95** | (1.11, 14.05) |
| DDT | 1.24 | (0.42, 3.64) | 1.13 | (0.39, 3.28) | 1.96 | (0,36, 3.96) | 1.73 | (0.50, 5.92) |
| Beta-HCH | 5.63** | (1.66, 19.01) | 1.91 | (0.51, 7.12) | 3.28 | (0.88, 12.27) | 2.08 | (0.54, 8.09) |
| PCB-118 | 2.23 | (0.73, 6.78) | 1.65 | (0.53, 5.15) | 1.5 | (0.44, 5.04) | 0.88 | (0.24, 5.04) |
|  |  |  |  |  |  |  |  |  |
| *Adjusted for Age and WHR  ** Values are significant at p<0.05 | | |  |  |  |  |  |  |

Supplementary Figure 1. Correlations of POPs Concentrations
